# Neuropsychological study of amyotrophic lateral sclerosis and parkinsonism-dementia complex in Kii peninsula, Japan

**DOI:** 10.1186/1471-2377-14-151

**Published:** 2014-07-21

**Authors:** Akihiro Shindo, Yukito Ueda, Shigeki Kuzuhara, Yasumasa Kokubo

**Affiliations:** 1 Department of Neurology, Mie University Graduate School of Medicine, Tsu, Mie, Japan; 2 Department of Rehabilitation, Mie University Hospital, Tsu, Mie, Japan; 3 Department of Medical Welfare, Faculty of Health Science, Suzuka University of Medical Science, Suzuka, Mie, Japan; 4 Kii ALS/PDC Research Center, Mie University, Graduate School of Regional Innovation Studies, Mie 514-8507, Japan

**Keywords:** Abulia, Apathy, Dementia, Amyotrophic lateral sclerosis, Parkinsonism–dementia complex

## Abstract

**Background:**

The Kii peninsula of Japan is one of the foci of amyotrophic lateral sclerosis and parkinsonism-dementia complex (ALS/PDC) in the world. The purpose of this study is to clarify the neuropsychological features of the patients with ALS/PDC of the Kii peninsula (Kii ALS/PDC).

**Methods:**

The medical interview was done on 13 patients with Kii ALS/PDC, 12 patients with Alzheimer’s disease, 10 patients with progressive supranuclear palsy, 10 patients with frontotemporal lobar degeneration and 10 patients with dementia with Lewy bodies. These patients and their carer/spouse were asked to report any history of abulia-apathy, hallucination, personality change and other variety of symptoms. Patients also underwent brain magnetic resonance imaging (MRI), single photon emission computed tomography (SPECT), and neuropsychological tests comprising the Mini Mental State Examination, Raven’s Colored Progressive Matrices, verbal fluency, and Paired-Associate Word Learning Test and some of them were assessed with the Frontal Assessment Battery (FAB).

**Results:**

All patients with Kii ALS/PDC had cognitive dysfunction including abulia-apathy, bradyphrenia, hallucination, decrease of extraversion, disorientation, and delayed reaction time. Brain MRI showed atrophy of the frontal and/or temporal lobes, and SPECT revealed a decrease in cerebral blood flow of the frontal and/or temporal lobes in all patients with Kii ALS/PDC. Disorientation, difficulty in word recall, delayed reaction time, and low FAB score were recognized in Kii ALS/PDC patients with cognitive dysfunction.

**Conclusions:**

The core neuropsychological features of the patients with Kii ALS/PDC were characterized by marked abulia-apathy, bradyphrenia, and hallucination.

## Background

The Muro district covers the mountainous areas of the southern coast of the Kii peninsula in Japan. Amyotrophic lateral sclerosis and parkinsonism-dementia complex (ALS/PDC) endemic to residents in this area is referred to as “Muro disease”. An epidemiological survey of the prevalence rates of amyotrophic lateral sclerosis (ALS) conducted in this area in the 1950s found very dense accumulation in two villages in particular, Hohara and Kozagawa. The prevalence rates in these villages were approximately 100 times those in other areas of Japan. Neuropathological features of ALS patients in this area (Kii ALS) were similar to those observed in the Chamorro people of Guam [1], and were characterized by a combination of neurofibrillary tangles (NFTs) in the brain and the changes of ALS [2]. Parkinsonism-dementia complex (PDC) is characterized clinically by progressive parkinsonism and dementia. This disorder is frequently superimposed with ALS clinically and neuronal loss with abundant NFTs in the temporal lobe, frontal lobe and brainstem without accompanying senile plaques was neuropathological hallmark [3,4].

Neuroradiological study with Kii ALS/PDC showed hypometabolism of frontotemporal lobes, with or without frontotemporal atrophy, resembling that of the frontotemporal lobar degeneration (FTLD), but different from Alzheimer’s disease (AD) [5]. Although neuroradiological study of Kii ALS/PDS was similar to FTLD, it is uncertain whether clinical features, especially in the characteristics of dementia, of Kii ALS/PDC are same as that of FTLD. Previous reports showed the clinical characteristics of Kii ALS/PDC were abulia and amnesia [6]. In the present study, we revealed abulia-apathy, hallucination, impairment of orientation, deterioration of recent memory and/or frontal executive dysfunction and bradyphreniaof patients with Kii ALS/PDC compared with patients with other types of dementia disorders, and used brain magnetic resonance imaging (MRI) and brain regional perfusion as assessed by single photon emission computed tomography (SPECT) to reveal the structural and functional abnormality linked to cognitive dysfunction in these patients.

## Methods

### Subjects

Thirteen patients with Kii ALS/PDC, 12 patients with AD, 10 patients with progressive supranuclear palsy (PSP), 10 patients with FTLD (behavior variant frontotemporal dementia type), and 10 patients with dementia with Lewy bodies (DLB) were enrolled in this study. The diagnosis of Kii ALS was made according to revised El Escorial [7] criteria since the clinical symptoms of Kii ALS are essentially the same as those of classical ALS and genetic tests excluded other types of ALS with gene mutation. The diagnosis of Kii PDC was made by a unique combination of levodopa-unresponsive parkinsonism and dementia, which are frequently accompanied by amyotrophy of the extremities [8]. Diagnosis of patients with AD, PSP and DLB was “probable” according to established diagnostic criteria [9-11], and the patients with FTLD were diagnosed by established diagnostic criteria [12]. One of 13 patients with Kii ALS/PDC was definitely diagnosed by autopsy [13]. Eleven of Kii ALS/PDC had moderate to severe parkinsonism with Hoehn-Yahr stage 3 to 5, and all of them were natives of Hohara village and its vicinity.

This study was approved by the ethics committee of the Mie University Hospital, Tsu, Mie, Japan. Written informed consent was obtained from all patients or their families prior to the study. The patients or next of kin provided written informed consent for the publication of individual clinical details as found in the descriptions of individual cases and Table 1.

**Table 1:** Summary of neuropsychological and neuroimaging data of patients with Kii ALS/PDC

								**RCPM**		**Verbal fluency**			
**Patient no.**	**Age**	**Sex**	**Diagnosis**	**Disease duration, y**	**Brain atrophy on MRI**	**Decrease in cerebral blood flow on SPECT**	**mmse (/30)**	**Total (/36)**	**Time (min sec)**	**Semantic**	**Letter**	**PAWLT (/10)**	**FAB (/18)**
1	64	M	Kii-ALS	Clinical	F	F, T	23	15	8 26	10	5	7	Not done
2	69	F	Kii-ALS	Clinical	T	F, T	22	25	7 14	5	1	5	10
3	68	M	Kii-PDC	Clinical	T	T, P	18	21	17 14	6	2	1	5
4	64	F	Kii-ALS/PDC	Clinical	F, T	F, T	12	20	16 26	10	4	2	Not done
5	66	F	Kii-PDC	Clinical	T	T, P	25	28	17 53	10	5	10	12
6	75	F	Kii-PDC	Clinical	T	F, T	25	21	12 30	11	5	6	13
7	76	F	Kii-PDC	Clinical	F, T	F, T	13	18	17 04	3	2	2	3
8	72	M	Kii-PDC	Autopsy	F, T	F, T	13	13	6 36	6	2	2	2
9	73	M	Kii-PDC	Clinical	F, T, P	F, T, P	12	5	11 37	7	2	2	Not done
10	76	M	Kii-ALS/PDC	Clinical	F, T	F, T	14	10	47 00	2	1	5	Not done
11	64	M	Kii-PDC	Clinical	F, T, P	F, T, P	16	24	7 24	7	5	3	12
12	68	F	Kii-PDC	Clinical	F, T	F, T	20	20	10 20	6	6	3	5
13	69	F	Kii-PDC	Clinical	F	F, P	17	17	18 20	8	7	5	0

*Abbreviations*: *MRI* magnetic resonance imaging, *SPECT* single-photon emission computed tomography, *MMSE* the Mini Mental State Examination, *RCPM* Raven’s Colored Progressive Matrices, *PAWLT* Paired-Associate Word Learning test, *FAB* the Frontal Assessment Battery, *F* frontal lobe, *T* temporal lobe, *P* parietal lobe, (-), absent/unremarkable.

### Inquiry to the carer/spouse and neuroimaging

Patients and their carer/spouse were asked to report any history of abulia-apathy, hallucination, personality changes (extraversion, neuroticism, openness to experience, agreeableness and conscientiousness), inappropriate social behavior/disinhibition, loss of empathy, loss of insight and abnormal eating behavior. The carers/spouses were not familiar with medical term, so we converted the medical term to easy words, such as apathy like a decrease of motivation, and were asked their impression regarding the patients. Any assessment instruments or quantitative scores were not used. We defined apathy as a disorder of motivation, and operationalized as diminished goal oriented behavior and cognition. In addition, we considered abulia as more severe type of apathy [14].

Neuroimaging studies of the head with magnetic resonance imaging (MRI) and angiography (MRA), and brain regional perfusion as assessed by single photon emission computed tomography (SPECT) were performed for all patients. All MRI studies were performed on either 0.5-T or 1.5-T units (FLEXART 0.5 T; Toshiba, Tokyo, Japan and Signa 1.5 T; GE Medical Systems, Milwaukee, WI). T1-and T2-weightedimages and fluid fluid-attenuated inversion recovery were obtained. We also used a SPECT scanner (model GCA-901A; Toshiba, Otawara, Tochigi, Japan) with a tracer technetium Tc99m ethyl cysteinate dimmer. The entire images were evaluated visually by several experienced neuroradiologists by blind manner. Statistical analysis was not done.

### Neuropsychological tests

All patients underwent a standard cognitive status assessment. ALS/PDC clinically show amnesia and bradyphrenia, therefore we chose Mini Mental State Examination (MMSE) [15] for global cognition, Raven’s Colored Progressive Matrices (RCPM) [16] for psychomotor speed, verbal fluency for frontal lobe function, the Paired-Associate Word Learning test (PAWLT) [17] for short-term memory and the Frontal Assessment Battery (FAB) [18] for the frontal lobe function as battery. The Standard Language Test of Aphasia (SLTA) [19] was used to evaluate language function and aphasia. Each patient underwent all neuropsychological tests on the same day. The Japanese version of the MMSE was used for overall assessment of cognition [15], and we compared the following: orientation (10 points), registration (3 points), attention and calculation (5 points), recall (3 points), and language and praxis (9 points). RCPM [16] was used to assess current intellectual function and psychomotor speed by measuring the time taken to perform a task. Although the motor impairment of the upper limb or dysarthria due to parkinsonism or ALS may affect the results of RCPM, in that case, we asked the patients to answer the number in oral, not by pointing at the number, or assisted them physically to avoid the effect of motor impairment to the utmost. To evaluate verbal fluency as a measure of language function, we asked patients to name as many items from a semantic category (animals) and a letter category (words beginning, representing Japanese mora) as possible for 1 minute. The lower limit of normal was set between 8 and 10 [20]. Short-term memory function was assessed by PAWLT. During this test, 10 word pairs were read to the patient in three trials, and the first of each pair was then presented for the patient to give the associated word [17]. Several patients were assessed with FAB [20]. All patients were checked for aphasia by a speech therapist, and we diagnosed the subtypes of aphasia according to the SLTA [19].

### Statistical analyses

The data are reported as mean ± SEM (standard error of the mean). All parameters were evaluated with Mann–Whitney U. The SPSS 22 software package was used to perform descriptive statistical analysis. Differences were considered significant when the p value was < 0.05.

## Results

### Representative cases

#### Case 2 (Kii ALS with dementia)

The patient was a 69-year-old woman born in the Kii peninsula with no family history of neurological diseases and no history of major illness. She presented initially with forgetfulness and reduced spontaneous motor and emotional behavior at age 67 years. The following year, she gradually developed dysarthria. At 69 years when she was admitted to our hospital, she presented with abulia-apathy, atrophy and fasciculation of the tongue, dysarthria, limb muscle atrophy, increased deep tendon reflexes and positive Babinski sign. Electromyography disclosed ongoing denervation muscle potentials in the biceps, the triceps, sternoclenocleidomastoideus, and the gastrocnemius. Her diagnosis was clinically definite ALS (revised El Escorial criteria [7]) at the time of admission. Her Score of the revised ALS Functional Rating Scale [21] was 34. Her MMSE score was 22 points, RCPM was 25 points and examination time was 7 minutes 14 seconds, verbal fluency was 5 (animal) and 1 (letter), PAWLT was 5 and FAB was10 points. SLTA did not present any aphasia detail as follows; auditory comprehension (9 of 10); spontaneous speech (5 of 6); naming (20 of 20); sentence repetition (5 of 5); kana word reading aloud (5 of 5); kanji word reading aloud (5 of 5); reading aloud short sentences (5 of 5); kana word comprehension (10 of 10); kanji word comprehension (9 of 10); reading comprehension (9 of 10); dictation of kana letters (10 of 10); dictation of kana words (5 of 5); dictation of kanji words (5 of 5) and dictation of short sentences (4 of 5). MRI scans revealed atrophy of the temporal lobes and SPECT images revealed a decline in cerebral blood flow in the temporal lobes. The clinical diagnosis was probable Kii ALS with dementia.

#### Case 8 (Kii PDC)

The patient was a 72-year-old man who was born in Hohara and had a history of hypertension. He had no family history about the neurodegenerative diseases. He developed resting tremor in his right hand and bradykinesia at 68 years. His parkinsonian symptoms of resting tremor and clumsy hands worsened progressively. At the age of 72 he suffered from visual hallucinations. After that, his verbal, motor and emotional behavior gradually became less spontaneous, and he was admitted to our hospital. Neurological examination at admission revealed parkinsonism with rigidity, bradykinesia, and postural instability, and dementia characterized by amnesia, abulia-apathy and bradyphrenia. His Hoehn Yahr scale was 5 [22]. MMSE score was 13 points, RCPM was 13 points and examination time was 6 minutes 36 seconds, verbal fluency was 6, PAWLT was 2. MRI scans revealed atrophy of frontal and temporal lobes and SPECT images revealed a decline in cerebral blood flow in the same lobes. When he died of pneumonia at age 74, an autopsy was performed after obtaining informed consent from his family. The formalin-fixed brain specimen weighed 1255 g., Gross inspection showed moderate atrophy of the hippocampus and striatum, severe depigmentation of the substantia nigra and locus coeruleus Microscopically, there were severe neuronal loss and many neurofibrillary tangles in the medial temporal lobe, nuclei of the brainstem, amygdala and nucleus of Mynert, and moderate neuronal loss of the anterior horn cells. Moderate α-synuclein pathology coexisted mainly in the nuclei of the brainstem. Neuropathological diagnosis was Kii PDC [13].

### Inquiry to the carer/spouse and neuroimaging

Clinical data of each group are shown in Table 2. Although the duration of the illness was significantly different, there was no significant difference in the duration of the cognitive impairment and education for Kii ALS/PDC, AD, PSP, and FTLD. Neuropsychological deficits developed in various time course in each patient. In some patients, neuropsychological deficits developed prior to motor symptoms, in other motor symptoms preceded neuropsychological deficits. The duration of disorder of patients with Kii ALS/PDC was longer than the patients with AD, PSP and FTLD. Abulia-apathy was prominent and present in 62% of Kii ALS/PDC patients. Among personality changes (extraversion, neuroticism, openness to experience, agreeableness and conscientiousness), inappropriate social behavior/disinhibition, loss of empathy, loss of insight and abnormal eating behavior, only decrease of extraversion was observed in Kii ALS/PDC. Hallucinations of the patients with Kii ALS/PDC were about well-formed complex vivid images of human faces or animals in the absence of the visual stimulates. Brain MRI showed atrophy of the frontal and/or temporal lobes, and SPECT revealed a decrease in cerebral blood flow of the frontal and/or temporal lobes in all patients with Kii ALS/PDC (Table 1). The results of neuroimaging tests are shown in Table 3.

**Table 2:** Clinical profiles of patients with Kii ALS/PDC and other types of dementia

	**Kii-ALS/PDC (n = 13)**	**AD (n = 12)**	**PSP (n = 10)**	**FTLD (n = 10)**	**DLB (n = 10)**
Sex (male/female)	6/7	4/8	6/4	4/6	4/6
Age at examination, y	69.5 ± 4.5	67.3 ± 5.4	71.3 ± 4.8	66.3 ± 9.0	68.9 ± 5.3
	(58–78)	(60–75)	(63–79)	(58–84)	(60–75)
Duration of the disorder, y	4.9 ± 1.6	2.5 ± 0.4*	2.0 ± 1.5*	2.4 ± 1.4*	3.3 ± 1.2
Duration of the cognitive impairement, y	2.6 ± 1.1	2.5 ± 0.4	2.0 ± 1.5	2.1 ± 1.2	2.4 ± 1.1
Education, y	9.9 ± 1.9	11.6 ± 3.2	10.7 ± 3.2	11.0 ± 2.6	11.5 ± 2.6
Clinical features (%)					
Halluciantion	38	0*	0*	0*	80*
Abulia-apathy	62	0*	50	30	30
Slowing of psychomotor speed	84	42	70	20*	70
Personality changes	77	58	80	80	60
Decrease of extraversion	77	50	80	80	60
Alteration of personarity	0	17*	10	70*	10
Inapproriate social behavior/disinhibition	0	8	0	30*	0
Aphasia	0	0	0	30*	0

*P < 0.05 compared with Kii ALS/PDC patients. *Abbreviations*: *ALS* amyotrophic lateral sclerosis, *PDC* parkinsonism-dementia complex, *AD* Alzheimer’s disease, *PSP* progressive supranuclear palsy, *FTLD* frontotemporal lobar degeneration, *DLB* dementia with Lewy bodies.

**Table 3:** Summary of neuroradiological data in patients with Kii ALS/PDC and other types of dementia

	**Kii-ALS/PDC (n = 13)**	**AD (n = 12)**	**PSP (n = 10)**	**FTLD (n = 10)**	**DLB (n = 10)**
MRI brain atrophy (%)					
Frontal	69.2	8.3	60.0	60.0	30.0
Temporal	84.6	58.3	0.0	80.0	0.0
Parietal	15.3	75.0	0.0	0.0	10.0
Occipital	0.0	0.0	0.0	0.0	0.0
SPECT decrease of cerebral blood flow (%)					
Frontal	84.6	16.7	80.0	90.0	30.0
Temporal	92.3	72.7	10.0	90.0	0.0
Parietal	30.7	83.3	0.0	20.0	70.0
Occipital	0.0	8.3	0.0	10.0	60.0

*Abbreviations*: *MRI* magnetic resonance imaging, *SPECT* single-photon emission computed tomography.

### Neuropsychological tests

The neuropsychological data of patients with Kii ALS/PDC are shown in Table 1 and that of each type of dementia are shown in Table 4. A significant difference in MMSE total scores was found between patients with Kii ALS/PDC and patients with AD and PSP despite the equivalent duration of the cognitive impairment in each group. MMSE also showed that patients with Kii ALS/PDC differed in their orientation score compared with AD and FTLD patients, and in their recall score compared with PSP and FTLD patients. Nor was there significant difference in the RCPM total score of each patient group, but patients with Kii ALS/PDC took significantly longer time to perform tasks than patients with AD and FTLD. Although the results of the verbal fluency test about a semantic category showed no significant difference between patient groups, there was a significant difference about in a letter category between Kii ALS/PDC and AD. The results of the PAWLT score was significantly lower in Kii ALS/PDC than in PSP patients. Although we examined FAB in not all, the results showed a trend that the scores of the patients with Kii ALS/PDC were worse than those of patients with AD.

**Table 4:** Summary of neuropsychological data in patients with Kii ALS/PDC and other types of dementia

	**Kii-ALS/PDC (n = 13)**	**AD (n = 12)**	**PSP (n = 10)**	**FTLD (n = 10)**	**DLB (n = 10)**
MMSE total score (/30)	19.2 ± 6.4	22.7 ± 1.2*	23.2 ± 2.5*	22.0 ± 4.5	20.7 ± 1.3
Orientation (/10)	5.8 ± 3.3	7.9 ± 0.6*	8.0 ± 1.8	8.0 ± 0.6*	6.8 ± 1.6
Registration (/3)	2.9 ± 0.3	3.0 ± 0.0	3.0 ± 0.0	3.0 ± 0.0	3.0 ± 0.0
Attention and calculation (/5)	1.8 ± 1.9	2.1 ± 1.5	1.6 ± 0.9	1.7 ± 1.4	1.5 ± 1.1
Recall (/3)	0.6 ± 0.9	1.1 ± 0.7	2.1 ± 1.0*	1.9 ± 0.9*	1.2 ± 1.1*
Language and praxis (/9)	7.9 ± 1.2	8.2 ± 1.1	8.5 ± 1.1	7.9 ± 0.9	8.1 ± 1.1
RCPM					
Total score (/36)	19.3 ± 6.8	22.3 ± 6.2	21.2 ± 4.1	22.0 ± 4.4	19.3 ± 6.8
Time (min sec)	14 37 ± 10 19	9 45 ±5 19*	12 56 ± 9 38	9 22 ± 3 51*	12 52 ± 6 39
Verbal fluency					
Semantic category	7.6 ± 3.2	8.5 ± 2.7	6.4 ± 2.9	7.8 ± 3.2	9.1 ± 3.2
Letter category	3.6 ± 2.0	6.9 ± 2.1*	5.1 ± 2.6	4.5 ± 2.2	4.4 ± 1.7
PAWLT (/10)	5.0 ± 3.2	5.4 ± 1.9	7.1 ± 1.6*	6.0 ± 2.8	6.5 ± 3.5
FAB (/18)	8.0 ± 5.5	13.9 ± 6.0*	12.0 ± 2.2	10.0 ± 2.2	12.3 ± 2.6
	(9 of 13 patients)	(8 of 12)	(4 of 10)	(4 of 10)	(3 of 10)

*p < 0.05, compared with Kii ALS/PDC patients.

## Discussion

The present study revealed that the core neuropsychological symptoms of patients with Kii ALS/PDC were abulia-apathy, hallucination, impairment of orientation, deterioration of recent memory and/or frontal executive dysfunction and bradyphrenia.

AD is a progressive neurodegenerative disorder that is characterized by the presence of amyloid deposits and NFTs together with the loss of cortical neurons and synapses [23]. The most profound and earliest cognitive deficits seem to be impairment of episodic memory and the ability to recall events that are specific to a time and place [24]. AD is characterized by deficits in attentional and executive functions [25]. Depression is the main psychiatric correlate of abulia-apathy in AD. Several studies have demonstrated a significant association between apathy and reduction in metabolic activity of the frontal lobes, and more severe parkinsonism suggesting that neuropathological changes in specific brain areas may underlie the high frequency of apathy in AD [26]. A recent study has shown that apathy is a behavioral marker of more aggressive dementia characterized by faster progression of cognitive impairment [27]. Delusions, and in particular paranoid delusions, are more common than hallucinations in AD [28].

PSP is one of the most common atypical parkinsonian syndromes. PSP pathology is characterized by the abnormal accumulation of tau protein accompanied by neuronal loss and gliosis, mainly in subcortical structures [29]. The striato-frontal dysfunction leads to dramatic deficits in planning, monitoring and recall, which evolves into dementia in PSP patients [30,31]. The cognitive impairment of PSP patients has been considered the archetype of subcortical dementia. The striking features are severe bradyphrenia, impaired verbal fluency, and difficulty with sequential actions or shifting from one task to another [32]. Cognitive tests that depend on visual performance are especially affected. Dementia is less severe than might be suggested by dysarthria, bradyphrenia, poor eye contact, and loss of facial expression. Abulia-apathy and disinhibition are common. Emotional incontinence is dominated by inappropriate weeping or, less frequently, laughing.

FTLD is the third most common cause of cortical dementia, following AD and DLB [12]. FTLD encompasses two major pathologic substrates that affect primarily thefrontal or temporal cortex, in some patients asymmetrically. Three prototypic neurobehavioral syndromes can result from FTLD. The most common clinical manifestations of FTLD are changes in character, disordered social conduct, and lack of affect, concern and insight, with relative preservation of memory function [33,34]. Cognitive deficits occur in the domains of attention, abstraction, planning, and problem solving, in keeping with a frontal dysexecutive syndrome, whereas primary tools of language, perception, and spatial functions are well preserved. Patients are not clinically amnesic. Two other prototypic clinical syndromes occur in FTLD: progressive non-fluent aphasia and semantic dementia [33]. The disorder of language occurs in the absence of impairment in other cognitive domains.

DLB was originally defined as a clinicopathologic entity with a specific constellation of clinical features, and a descriptive approach was proposed for assessing neuropathology [9]. The only neuropathologic requirement for DLB is the presence of Lewy bodies somewhere in the brain of a patient with a clinical history of dementia. Recurrent, complex visual hallucinations continue to be one of the most useful signposts to a clinical diagnosis of DLB. Disability in DLB derives not only from cognitive impairment but also from neuropsychiatric, motor, sleep, and autonomic dysfunction. The cognitive profile of DLB comprises both cortical and subcortical impairments with substantial attentional deficits and prominent executive and visuospatial dysfunction [35,36].

In this study, the clinical features of Kii ALS/PDC are abulia-apathy and hallucination, but not alteration of personality and aphasia. The patients with Kii ALS/PDC share some part of symptoms with PSP and DLB patients. In MMSE and PAWLT, the patients with Kii ALS/PDC showed more severe disorientation than those with AD and FTLD, and more severe deterioration of recent memory than those with PSP and FTLD despite the equivalent duration of the cognitive impairment in each group. Recall performance can potentially be impaired as a result of a primary amnesia, or secondarily as a result of poor frontal executive function, therefore it was difficult to discriminate a primary amnesia and poor frontal executive function in the patients with Kii ALS/PDC in this study. RCPM results revealed that the bradyphrenia observed in Kii ALS/PDC patients was even slower than that observed in AD patients and FTLD patients. Although the results of FAB, which was examined only in several selected cases, showed the patients with Kii ALS/PDC might have frontal executive dysfunction, there was a limitation of the study in view of the central importance of assessing frontal lobe functions in this patient group.

Most characteristic cognitive deficit of Kii ALS/PDC was abulia/apathy. Abulia/apathy may reflect neuronal loss and tau pathology especially in the anterior cingulate gyrus in which the decrease of the cerebral blood flow was also detected in SPECT (unpublished data). Visual hallucination may be linked to α-synuclein pathology [37]. The patients with Kii ALS/PDC lacked the typical symptoms of behavior variant FTD, semantic dementia and non-fluent progressive aphasia, those were personality change, inappropriate social behavior/disinhibition, loss of empathy, loss of insight, abnormal eating behavior and aphasia, except for decrease of extraversion, apathy and reduced speech. Although Kii ALS/PDC showed atrophy and the decrease of the blood flow in the frontal lobe and temporal lobe, the neuropsychological symptoms of patients with Kii ALS/PDC was different from those of the patients with FTLD. Also Kii ALS/PDC showed unique cognitive impairments comparing with AD, PSP, FTLD and DLB.

This study has a limitation in that we could not evaluate focus on frontal function except for FAB, none of tests are specifically designed for patients with ALS. And the motor impairment due to parkinsonism or ALS may affect the results of battery. There is a possibility of the effect to the performance in the test. We have only small cases of Kii ALS/PDC, and statistical comparison also has a limitation. However, the results of clinical symptoms, neuropsychological tests and neuroradiological tests seemed to be mutually related. These results are expected to contribute something to solving Kii ALS/PDC.

## Conclusion

Cognitive impairment of Kii ALS/PDC patients is unique frontal-subcortical and temporal-cortical dementia.

## Abbreviations

ALS/PDC: Amyotrophic lateral sclerosis and parkinsonism-dementia complex; ALS: Amyotrophic lateral sclerosis; PDC: Parkinsonism-dementia complex; MRI: Magnetic resonance imaging; SPECT: Single photon emission computed tomography; AD: Alzheimer’s disease; PSP: Progressive supranuclear palsy; FTLD: Frontotemporal lobar degeneration; DLB: Dementia with Lewy bodies; MMSE: Mini Mental State Examination; RCPM: Raven’s Colored Progressive Matrices; PAWLT: Paired-Associate Word Learning Test; FAB: Frontal Assessment Battery; SLTA: Standard Language Test of Aphasia.

## Competing interests

The authors declare no conflicts interest regarding this manuscript.

## Authors’ contributions

AS carried out conception and design of study, analysis and interpretation of data, collection and assembly data, and draft of the article. YU carried out the analysis of neuropsychological tests. SK conceived of the study, and participated in its design and coordination and helped to draft the manuscript. YK carried out critical revision of the article for important intellectual content, and final approval of this article. All authors read and approved the final manuscript.

## Pre-publication history

The pre-publication history for this paper can be accessed here:

http://www.biomedcentral.com/1471-2377/14/151/prepub
